# Identification of Type 2 Diabetes Management Mobile App Features and Engagement Strategies: Modified Delphi Approach

**DOI:** 10.2196/17083

**Published:** 2020-09-11

**Authors:** Hanan A Alenazi, Amr Jamal, Mohammed A Batais

**Affiliations:** 1 National Health Information Center Riyadh Saudi Arabia; 2 Family and Community Medicine Department College of Medicine King Saud University Riyadh Saudi Arabia; 3 Research Chair of Evidence-Based Healthcare and Knowledge Translation King Saud University Riyadh Saudi Arabia

**Keywords:** diabetes, mobile features, engagement strategies, mobile app, Delphi consensus

## Abstract

**Background:**

Diabetes is a significant public health issue. Saudi Arabia has the highest prevalence of type 2 diabetes mellitus (T2DM) in the Arab world. Currently, it affects 31.6% of the general population, and the prevalence of T2DM is predicted to rise to 45.36% by 2030. Mobile health (mHealth) offers improved and cost-effective care to people with T2DM. However, the efficiency of engagement strategies and features of this technology need to be reviewed and standardized according to stakeholder and expert perspectives.

**Objective:**

The main objective of this study was to identify the most agreed-upon features for T2DM self-management mobile apps; the secondary objective was to identify the most agreed-upon strategies that prompt users to use these apps.

**Methods:**

In this study, a 4-round modified Delphi method was applied by experts in the domain of diabetes care.

**Results:**

In total, 11 experts with a mean age of 47.09 years (SD 11.70) consented to participate in the study. Overall, 36 app features were generated. The group of experts displayed weak agreement in their ranking of intervention components (Kendall W=0.275; *P*<.001). The top 5 features included insulin dose adjustment according to carbohydrate counting and blood glucose readings (5.36), alerting a caregiver of abnormal or critical readings (6.09), nutrition education (12.45), contacts for guidance if required (12.64), and offering patient-specific education tailored to the user’s goals, needs, and blood glucose readings (12.90). In total, 21 engagement strategies were generated. Overall, the experts showed a moderate degree of consensus in their strategy rankings (Kendall W=0.454; *P*<.001). The top 5 engagement strategies included a user-friendly design (educational and age-appropriate design; 2.82), a free app (3.73), allowing the user to communicate or send information/data to a health care provider (HCP; 5.36), HCPs prescribing the mobile app in the clinic and asking about patients’ app use compliance during clinical visits (6.91), and flexibility and customization (7.91).

**Conclusions:**

This is the first study in the region consisting of a local panel of experts from the diabetes field gathering together. We used an iterative process to combine the experts’ opinions into a group consensus. The results of this study could thus be useful for health app developers and HCPs and inform future decision making on the topic.

## Introduction

Type 2 diabetes mellitus (T2DM) is one of the leading causes of disability and mortality worldwide, creating a substantial economic burden on health systems and social well-being [1,2]. In Saudi Arabia, studies evaluating T2DM prevalence showed a continuously increasing trend, reaching an age-adjusted prevalence of 31.6%, which is considered among the highest worldwide [3,4].

Diabetes is a chronic, complex syndrome requiring condition-related knowledge and self-management skills to optimize glycemic control and improve health outcomes. Mobile apps could be helpful tools for supporting chronic disease screening, enhancing the ability of people with diabetes to manage the disease, and ensuring their easy accessibility to health care services. These apps boost the opportunity to increase health care access for vulnerable populations. The apposite use of mobile health (mHealth) offers improved and cost-effective care to people with T2DM through improved diabetes self-management [5-7].

Globally, the annual growth in the penetration rate of internet and mobile phone use is estimated to be 13%. In Saudi Arabia, which has a total population of 33.25 million, 30.25 million (91%) are internet users, 25 million (75%) are active social media users, and there are 56.80 million (171%) mobile subscriptions [8]. However, the utility features of this technology need to be standardized and reviewed according to stakeholder and expert perspectives [9].

Several systematic reviews, meta-analyses, and meta-regression analyses found that digital or telemedicine interventions could be more effective in enhancing the outcomes of managing T2DM compared to standard care. The apps seem to increase the perception of self-care by contributing to better knowledge among people with T2DM. People with T2DM also become more confident in dealing with their illness, primarily due to a decrease in fears resulting from a lack of information; for example, they develop more confidence in how they should deal with potential hypoglycemic episodes [10-14]. Another systematic review, on the other hand, claims that there is limited evidence supporting the effectiveness of diabetes apps [15].

A systematic review that evaluated free mobile apps for control and self-management of type 1 diabetes mellitus (T1DM) available in 2015 found that 56 of these apps did not even meet the minimum requirements or work efficiently. While there was a significant number of available apps, the study results indicated that only 9 of the 65 reviewed mobile apps are adaptable and useful for self-management [16]. Most insulin dose calculator apps provide incorrect or inappropriate dosage recommendations, which puts patients at risk. Therefore, proficient health care providers (HCP) should be involved in the app design stage to address safety during self-management and health education [17]. There is a need for comprehensive, efficient, and flexible mobile apps for self-management of diabetes with more features to increase the number of long-term users and influence better self-management [16,17] and patient empowerment.

A study of several focus groups concerning patient perspectives and expectations of diabetes self-management and eHealth found that there were significant variations in the health care services experienced by patients with diabetes, and none of the evaluated services met all users' needs [18]. Patient groups seem to differ in expectations and needs concerning self-management and eHealth for self-management purposes. In a Dutch study, several participants worried about the implementation of eHealth being a consequence of budget cuts in care [19]. Therefore, we chose to engage experts and to use formal consensus methods that have been used to guide action in areas of research in which there is inconsistent or contradictory scientific evidence. Consensus techniques are an efficient and valid method for identifying and collecting information on a subject on which there is little evidence or agreement. Consensus techniques allow one to obtain quantitative estimates from qualitative strategies and to determine the degree of agreement among participants [20].

Delphi techniques were developed in the 1950s. They are a structured and multistage process in which a panel of experts is invited to be a part of a series of rounds to identify and achieve consensus on a specific issue. The consensus is sought through information feedback and iteration in the form of rounds and phases. The process is terminated when a consensus is reached. The anonymity offered by the Delphi method can reduce the inhibition that usually occurs during decision-making; individuals are more open in their answers when their answers are deidentified. The Delphi technique is useful when working with highly subjective elements in which it is difficult to determine their intrinsic value [21,22]. The Delphi technique is a unique method used to gain consensus from experts who hold varied viewpoints or professional experiences. This technique can be used to build agreement and allow groups to judge frameworks [23,24].

The main objective of this study was to identify the most agreed-upon features for T2DM self-management mobile apps. The secondary objective is to identify the most agreed-upon strategies that encourage users to use these apps. Our study aimed to determine an expert panel’s consensus opinion on two questions:

1. What are the diabetes management components that diabetes experts agree are most likely to be effective when delivered via a mobile app?
2. What are the engagement strategies that diabetes experts believe are the most likely to be effective for diabetes management when using a mobile app?

## Methods

### Study Design

A 4-round modified Delphi study was conducted to identify mobile app features and engagement strategies for T2DM self-management. There were three reasons for choosing the Delphi technique: (1) the Delphi technique has been useful for measuring group consensus concerning medical information technology when used in health care; (2) there was no established evidence regarding self-management of T2DM or engagement strategies in the context of developing mobile apps, and (3) the outcomes of Delphi decisions focus on decision making in fields that are strongly susceptible to change. Individual decision makers have more influence than those with underlying rules [20,25]. We used supplemental techniques to ensure the validity of study outcomes by creating a steering committee that includes subject experts to oversee the design, execution, and analysis of all study phases. An agreement between the steering committee members was reached by discussion to approve the participant selection, consensus threshold, survey format and questions, and analysis process; in addition, they wrote down comments and discussed any open points during all 4 rounds. Thus, a modified Delphi technique was used to identify mobile app features and engagement strategies for T2DM self-management.

### Participants

In total, 11 multidisciplinary experts were purposively selected from a range of scientific networks. Their professions included family physician, clinical informatician, diabetologist, clinical pharmacist, endocrinologist, exercise physiologist, nurse diabetes educator, health educator, clinical dietitian, consultant in medical education, and psychologist. Previous literature reports have indicated that the Delphi technique does not require a particular sample size. The minimum number of participants should be at least 7, although 10 to 20 are advisable [26,27]. A purposive sample is necessary to ensure the variability of the invited experts’ background and experience. The research project steering committee used their expertise to judge the suitability of the invited experts. After identifying the experts, formal contact was established through email, phone, or an in-person meeting. The experts were also asked whether they could further recommend experts who could add value to the project, as a snowball sampling technique. All of the experts who were approached agreed to participate in the project. The experts were all working in Saudi Arabia. Detailed profiles of the respondents are provided in Table 1. The selection of experts participating in the study was based on their diverse professional backgrounds and experience related to the care of people with T2DM. Apart from the clinical informatician, knowledge related to diabetes apps was not one of the selection criteria. Having a similar professional background and experience of a preexisting participant was the only exclusion criteria for the study.

**Table 1:** Profiles of the expert panel members (N=11).

Professional title	Latest academic degree	Specialized fields	Years of experience in health care	Years of experience in diabetes care	Number of patients per week	Years of experience in health informatics research	Years of experience in diabetes research	Gender
Professor, public health researcher, consultant in family and community medicine	FRCGP	Diabetes prevention and management, primary care, public health, evaluation research, health services research, health promotion, health education, patient-centered care design	33	33	18	0	33	Male
Professor, consultant in family and community medicine, researcher	Diploma in Medical Education	Primary care research, patient-physician decision making, interprofessional education, public health research, medical education, knowledge translation, communication skills	32	32	14	2	32	Male
Assistant professor, researcher, consultant diabetologist	Fellowship in diabetes	Diabetes prevention and management (tertiary care), clinical trials, population health research, patient education, community participation	10	7	70	0	6	Male
Endocrinologist consultant, president of Saudi Society of Endocrinology and Metabolism	SBIM	Health services (not for profit), diabetes prevention and management (tertiary care), metabolic diseases	30	30	45	0	30	Male
Assistant professor, consultant, Director of King Abdullah Arabic Health Encyclopedia	Diploma in research methodology	Health care information technology, informatics, health care management, medical education, patient safety, clinical research, public health, telehealth	17	17	0	10	1	Male
Nurse educator, head of the education department	BSc	Patient education, diabetes prevention and self-management, clinical research	25	25	100	0	15	Female
Health education specialist	BSc	Health education, diabetes prevention and self-management, primary and tertiary care	5	3	50	0	1	Female
Consultant psychologist	Psychology fellowship	Psychology care, diabetes psychology prevention management care, clinical research, communication skills	10	5	10	0	5	Male
Senior clinical dietitian	MSc	Clinical research, diabetes nutrition prevention management care, primary and tertiary care	6	3	30	0	3	Female
Assistant professor, clinical pharmacist	PhD	Clinical trials, clinical pharmacist, patient education, patient adherence	19	7	8	0	4	Female
Assistant professor, consultant physiology	PhD	Diabetes prevention and management, primary and tertiary care	21	3	20	0	3	Male

### Selection of the Appropriate Expert Panel

Experts were selected from hospitals, academic institutes, and associations in Saudi Arabia. Moreover, we also invited those who had participated in recent regional diabetes conferences. The inclusion criteria were comprised of 3 parameters: (1) clinicians or researchers with expertise in diabetes care, (2) who possessed at least 3 years of experience in the diabetes care field, and (3) who were willing to participate.

### Steering Committee

This study included steering committee that was comprised of a diabetes educator; a health informatics specialist and consultant in family medicine and medical informatics; and a consultant in family medicine and diabetologist. The committee oversaw the design, execution, and analysis of all phases of this study. An agreement was reached by discussion to approve the participant selection, consensus threshold, survey format and questions, and analysis process.

### Delphi Process

**Figure 1:**
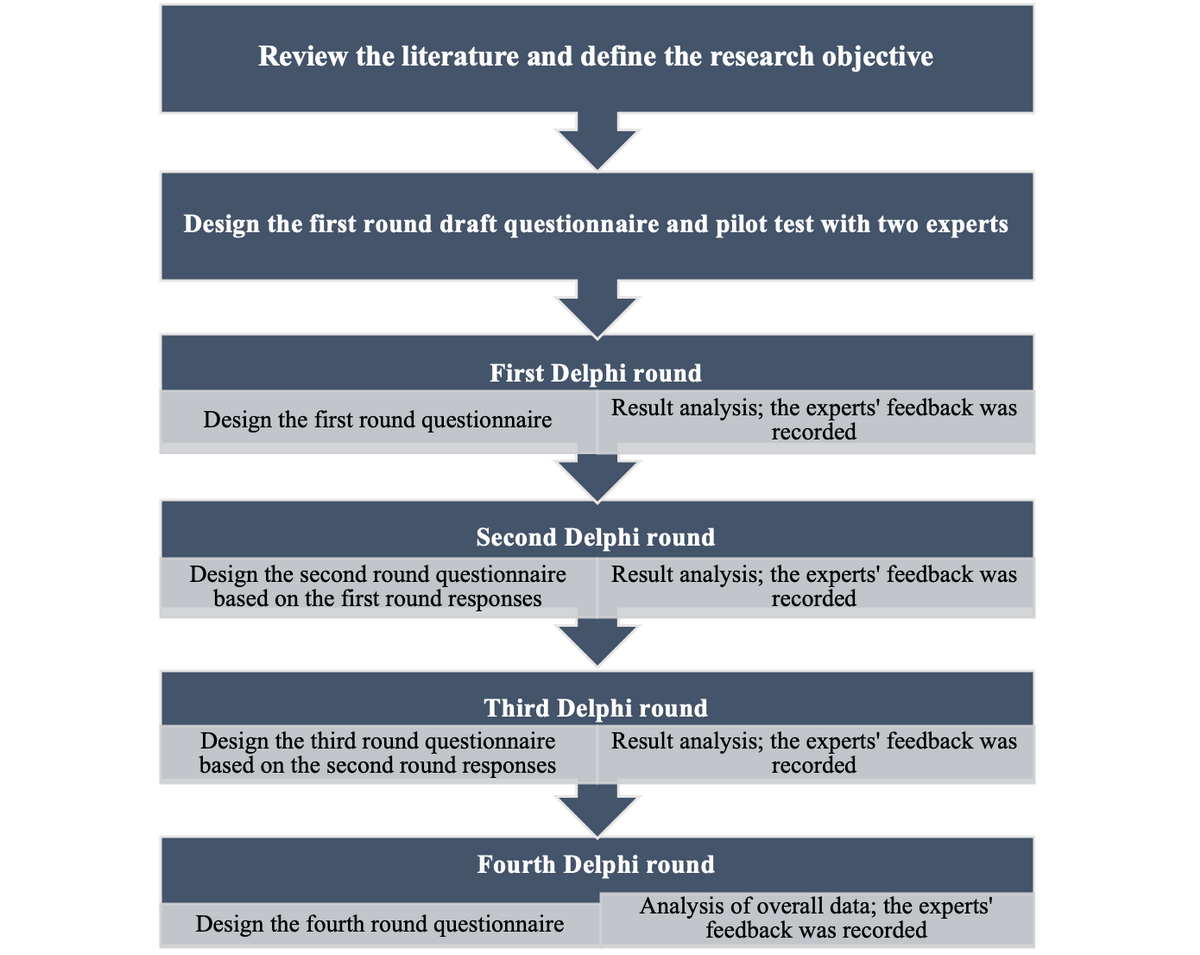
The modified Delphi process followed in this study.

A minimum of 3 survey rounds were prospectively planned; rounds would continue until a consensus agreement was reached (Figure 1). The Delphi process started in November 2017 and ended in June 2018. Each round was conducted over 5 weeks, starting with the distribution of the materials. A reminder email was sent 1 week and 24 hours before the closure of the round, and repeated until the experts responded to the survey. The results generated from the previous round would be used in each subsequent round. For data collection in the first round, a questionnaire was sent via email. From the second round onward, an electronic survey instrument (SurveyMonkey) was used to enable efficient and timely data collection from the participants.

### Pilot Study

The primary draft survey was pilot tested by two experts with expertise in diabetes research who not included in the original study. Pilot testing determined the time it took to complete the survey and whether the survey wording was clear. After minor amendments arising from the pilot testing, the first round survey format was considered complete.

### First Round

By using a survey with open-ended questions, the first round aimed to generate a list of items. Participants were asked to consider which items should be included when creating a mobile app designed to help people with diabetes self-manage their illness. The initial survey consisted of three sections: (1) a brief overview of the Delphi process; (2) demographic information; (3) two open-ended questions asking participants which items should be included when designing the mobile app. The experts were invited to provide at least three responses to each question:

1. Which features or components of management care delivered by the mobile app would be most effective in helping patients with diabetes improve their health?
2. What are the best strategies or techniques that should be used to engage people with diabetes to use such a mobile app?

Each question was preceded with the following statement: “Please answer the questions below based on your clinical experience, knowledge of the guidelines, and relevant theories. Please provide at least 3 responses for each question.”

### Second Round

In the second round, a checklist was generated from the first round results submitted by the experts, and they were asked their opinion. Furthermore, they were also asked to accept, delete, or modify each item. Qualitative responses offered insight into the differences in experts’ perspectives. The items had been modified via qualitative feedback.

### Third Round

In the third round of the Delphi process, participants were invited to rate the importance of each item of the alphabetically ordered checklist generated from the first and second rounds. They were asked to rate their agreement with each of these techniques for the 3 different questions on a 5-point Likert-scale. The scale consisted of 5 responses: 1=strongly disagree, 2=disagree, 3=neither agree nor disagree, 4=agree, and 5=strongly agree, as well as the option, “I do not know.”

### Fourth Round

Experts were provided with the items listed alphabetically, alongside the mean of agreement ratings. Experts were asked to rank their responses from 1 (most likely to be the best) to n=the total number of items (least likely to be the best) for each question. At this stage, participants were only asked to rank responses for which there had been broad agreement in previous rounds. At this point, there was the option to make final comments, if any.

### Data Analysis

#### Data Management 

Quantitative data analyses were performed with SPSS (version 20.0; IBM Corp). The descriptive statistics for each item were calculated, including the mean agreement scores, standard deviation, and the Kendall W statistic which employs the chi-square test to test the independence of the component rankings.

#### Feedback

Based on Research and Development Corporation (RAND) recommendations regarding participant feedback, the findings from the previous round were compiled for Rounds 2, 3, and 4 [28]. The feedback report was provided to participants 2 weeks before the initiation of the next round. The feedback from the last round was offered to participants 1 month after the round ended.

#### First-Round Analysis

After the first round, the total number of completed questionnaires was 11. For each question, two individuals from the steering committee assessed, reviewed, and summarized the results of the first round independently. Similar ideas were clustered together into emerging components. Duplicate responses were merged. Responses that were not related to the study objectives, such as “consume high fiber diet through vegetable and fruit,” were deemed not applicable and they were removed. The two reviewers met to discuss any differences arising from these independent analyses. Subsequently, the results were reviewed, and a discussion was held to validate the results and categorize them into domains.

#### Second-Round Analysis

The total number of completed questionnaires was 11. After all responses were received, the steering committee compared the responses and reviewed the proposals to generate the final list.

#### Third-Round Analysis

The total number of completed surveys was 11. Participants were asked to rate their agreement with the 36 mobile app features and 21 engagement strategies mentioned in the first and second rounds on a 5-point Likert scale. Descriptive statistics for each item were calculated, including the mean agreement scores and standard deviation.

#### Fourth-Round Analysis

In the final round, participants were asked to rank the mobile features from 1 (highest) to 36 (lowest). The value of the 36 features was determined in the first and second rounds. Moreover, participants were asked to rank each engagement strategy from 1 (highest) to 21 (lowest). The value of the 21 engagement strategies was determined in the first and second rounds. Descriptive statistics for each item were computed, including the mean ranking scores and standard deviation (SD). We used the Kendall W coefficient of concordance, which measures the extent to which judges agree on their rankings of items. The Kendall W statistic uses the chi-square test to investigate the independence of the components’ rankings.

### Ethical Considerations

Ethical approval to conduct this study was obtained from the King Saud University Institutional Review Board (number E-17-2608). All participants were asked to sign a consent form prior to starting the rounds. Experts were asked to create a list of the best features and engagement strategies, which were subsequently rated and ranked anonymously to avoid biases.

## Results

### Demographic Characteristics of Participants

A total of 11 experts consented to participate in the study. They had a mean age of 47.09 years (SD 11.70; range 28-62). In total, 4 rounds of Delphi exercises were completed over 6 months, and all of the expert participants (n=11) completed all 4 rounds.

### Features

A total of 77 features were generated in response to the first question, “what are the diabetes management components that diabetes experts agree are most likely to be effective when delivered via an app,” with an average of 7 features mentioned per participant in the first and second rounds (Multimedia Appendix 1). Duplicate features were removed, and some were merged (n=36), and the resulting 36 features were categorized into 5 domains (Table 2). No further items were added after rounds 3 and 4 for the whole list of items. Of the 36 features, 11 had a high mean agreement rating (above 4.50). These features were “a reminder for the health care providers' appointments, screening, and routine lab tests” (4.73), “carbohydrate counter” (4.72), and “diet planning” (4.72). All of the next 6 features scored the same (4.64): “nutrition education,” “contacts for guidance if needed,” “offer patient-specific education tailored to the user’s goals, needs, and blood glucose readings,” “find the nearest urgent health care services/centers,” “database for local restaurants and stores providing diabetes-friendly foods,” and “providing Arabic and English versions”. Both “medication reminders by notifications” and “the synchronization with the system, syncing with electronic medical records/personal health records, syncing with glucometers, and continuous glucose monitors or insulin pumps” scored the same (4.54). Overall, the original group of experts displayed weak agreement (Kendall W=0.275) in their ranking of intervention components (*χ*^2^_35_=106.017, *P*<.001).

There were 5 top features: (1) adjusting insulin doses according to carbohydrate counting and blood glucose readings (5.36); (2) alert caregiver (such as by text message) for abnormal or critical readings (6.09); (3) nutrition education (12.45); (4) contacts for guidance if required (12.64); and (5) offering patient-specific education tailored to the user’s goals, needs, and blood glucose readings (12.90).

**Table 2:** Responses generated by the expert group concerning the features.

Rank	Responses generated^a^	Domain	Agreement rating^b^				Ranking score^c^	
			Mean (SD)	Mode	Agree: disagree^d^	Mean (SD)		Mode
1	Reminder for the health care providers’ appointments, screening, routine lab tests, etc	Follow-up care	4.73 (0.47)	5	11:0	13.00 (7.58)		14
2	Carbohydrate counter and health diet planning	Nutrition and diet management	4.72 (0.47)	4	11:0	13.73 (7.72)		12
3	Nutrition education	Nutrition and diet management	4.64 (0.50)	5	11:0	12.45 (8.51)		3
4	Contacts for guidance if needed	Follow-up care	4.64 (0.50)	5	11:0	12.64 (12.22)		1
5	Offer patient-specific education tailored to the user’s goals, needs, and blood glucose readings	Education	4.64 (0.50)	5	11:0	12.90 (11.54)		5
6	Find the nearest urgent health care services/centers	Follow-up care	4.64 (0.50)	5	11:0	15.18 (10.33)		12
7	Database for local restaurants and stores providing diabetes-friendly foods	Nutrition and diet management	4.64 (0.50)	5	11:0	19.45 (9.09)		25
8	Providing Arabic and English versions	Mobile design and features	4.64 (0.67)	5	10:1	21.36 (12.47)		29
9	Medication reminders by notifications	Medication	4.54 (0.69)	5	10:1	15.09 (7.28)		7
10	Syncing with electronic medical record/personal health record	Mobile design and features	4.54 (0.52)	5	11:0	24.82 (8.67)		32
11	Syncing with meters, continuous glucose monitors, or insulin pumps	Mobile design and features	4.54 (0.52)	5	11:0	27.18 (6.40)		26
12	Alert caregiver (eg, by SMS text message) for abnormal or critical readings	Home monitoring	4.45 (0.52)	4	11:0	6.09 (5.16)		2
13	Blood glucose monitoring diary; give results for averages in graphs	Home monitoring	4.45 (0.69)	5	10:1	14.64 (8.32)		9
14	Individualized exercise suggestions or prescriptions	Physical activity	4.45 (0.69)	5	10:1	14.82 (6.92)		13
15	Assessing medication adherence	Medication	4.45 (0.52)	4	11:0	15.73 (10.46)		6
16	Online consultation (communication and patient monitoring) by health care providers	Follow-up care	4.45 (0.69)	5	10:1	17.18 (8.54)		17
17	Prescription refill reminders	Follow-up care	4.45 (0.52)	4	11:0	17.73 (8.94)		16
18	Special occasion management during fasting, Hajj, sick days, and travel	Education	4.45 (0.69)	5	10:1	21.18 (9.63)		7
19	Medication reconciliation (a comprehensive list of medications should include all prescribed medications, herbals, vitamins, etc)	Medication	4.45 (0.69)	5	10:1	21.27 (8.95)		19
20	Create, view, and manage alerts for personalized target goals	Education	4.36 (0.50)	4	11:0	17.64 (9.32)		10
21	General education (such as foot care, wound care, dental care, psychiatric symptoms associated with diabetes, driving, how to use the glucometer)	Education	4.36 (0.81)	5	9:2	19.09 (10.23)		11
22	Database for traditional low-carbohydrate recipes and traditional food carbohydrates and calories	Nutrition and diet management	4.36 (0.92)	5	10:1	20.09 (10.14)		4
23	Sharing blood glucose readings with health care providers	Home monitoring	4.36 (0.50)	4	11:0	21.54 (8.64)		10
24	Activity sensor trackers (such as step counters) or integration with wearable trackers	Physical activity	4.27 (0.65)	4	10:1	17.18 (9.73)		6
25	Reminders to check blood glucose levels and ketones if needed	Home monitoring	4.27 (0.47)	4	11:0	17.18 (9.58)		3
26	Food barcode scanner and meal picture detection to log food	Nutrition and diet management	4.27 (0.65)	4	10:1	19.00 (9.89)		6
27	Workouts and exercise demonstrations	Physical activity	4.27 (0.65)	4	10:1	24.54 (11.58)		33
28	Find the nearest health and fitness activities	Physical activity	4.18 (0.87)	4	10:1	24.36 (10.13)		34
29	Pill identifier, drug list name with detailed information	Medication	4.18 (1.33)	5	8:3	25.73 (11.05)		36
30	Integrating the app with common social media platforms	Social media and communication	4.18 (0.75)	4	9:2	27.45 (7.05)		26
31	A food diary to track meals	Nutrition and diet management	4.09 (0.70)	4	9:2	20.82 (8.33)		19
32	Using anxiety and depression scales and providing customized advice to see health care providers	Psychosocial care	4.09 (0.70)	4	9:2	30.00 (7.17)		29
33	Adjusting insulin doses according to carbohydrate counting and blood glucose readings	Medication	4.00 (0.77)	4	8:3	5.36 (6.47)		5
34	Allowing for chat services for communication between users	Social media and communication	4.00 (0.77)	4	10:1	21.54 (10.00)		8
35	Body measurement trackers	Nutrition and diet management	3.91 (0.94)	4	8:3	22.18 (7.92)		15
36	Alert caregiver (such as by SMS text message) for missed doses	Home monitoring	3.82 (0.98)	4	9:2	15.81 (11.98)		8

^a^The features are ordered in terms of the mean agreement score from round 3.

^b^Agreement rating, where 1=strongly disagree and 5=strongly agree.

^c^Ranking score, where 1=highest and 36=lowest.

^d^Agree: disagree is the ratio of “agree” and “strongly agree” to “neither,” “disagree,” and “strongly disagree,” which was used as an inclusion criterion for round 3.

### Engagement Strategies

A total of 53 engagement strategies were generated in response to the second question, “what are the engagement strategies that diabetes experts believe are the most likely to be effective in diabetes management when using a mobile app.” There were an average of 3.5 strategies suggested per participant in the first and second rounds (Multimedia Appendix 2). After the merging of similar strategies, the number of engagement strategies was reduced to 21 (Table 3). The resultant 21 strategies were categorized into 5 domains. No further strategies were added after Rounds 3 and 4 for the whole list of strategies. Out of the 21 engagement strategies, 4 had a high mean of agreement rating (above 4.40): (1) the app should be a free app (4.64); (2) allow the user to communicate or send information/data to a health care provider (4.54); (3) a user-friendly design (such as educational and age-appropriate design; 4.45); and (4) flexibility and customization (4.45). Overall, the experts showed a moderate degree of consensus in their ranking of the strategies (Kendall W=0.454, *χ*^2^_20_=99.924, *P*<.001).

The top 5 engagement strategies included several parameters: (1) a user-friendly design (such as an educational and age-appropriate design; 2.82); (2) the app should be free (3.73): (3) allowing the user to communicate or send information/data to a health care provider (5.36); (4) health care providers prescribing the mobile app in the clinic and asking about the patients’ app use compliance during clinical visits (6.91), and (5) flexibility and customization (7.91).

**Table 3:** Responses generated by the expert group concerning engagement strategies.

Rank	Responses generated^a^	Domain	Agreement rating^b^				Ranking score^c^	
			Mean (SD)	Mode	Agree: disagree^d^	Mean (SD)		Mode
1	The app should be free	Cost	4.64 (0.67)	5	10:1	3.73 (3.35)		2
2	Allowing the user to communicate or send information/data to health care providers	Communication or support	4.54 (0.522)	5	11:0	5.36 (3.04)		8
3	A user-friendly design (such as educational and age-appropriate design)	Easy to use	4.45 (0.52)	4	11:0	2.82 (2.64)		1
4	Flexibility and customization	Easy to use	4.45 (0.52)	4	11:0	7.91 (6.92)		4
5	Cross-platform device syncing (sync between mobile and other devices)	Features	4.36 (0.67)	4	10:1	13.73 (5.60)		16
6	Allowing chat services so users can communicate and support each other	Communication or support	4.27 (0.47)	4	11:0	9.45 (5.28)		12
7	Taking feedback and adding new features	Communication or support	4.27 (0.47)	4	11:0	10.00 (4.86)		8
8	Having health coaches explain the usefulness of the app through health campaigns	Advertisement	4.27 (0.65)	4	10:1	10.36 (4.94)		10
9	Using accessibility features (such as text-to-speech for people with limited vision)	Features	4.27 (0.65)	4	10:1	11.00 (4.19)		12
10	Using varying teaching methods (such as audiovisual, illustration, alarm)	Communication or support	4.18 (0.75)	4	9:2	13.18 (5.88)		13
11	Allowing the users to share their progress with family, friends, others through integration with common social media platforms	Communication or support	4.18 (0.60)	4	10:1	13.45 (5.94)		17
12	Syncing with electronic medical records/personal health record	Features	4.18 (0.60)	4	10:1	13.45 (4.27)		20
13	Having the official websites and social media accounts of scientific associations and organizations recommend the use of the app	Advertisement	4.09 (0.54)	4	10:1	14.82 (4.12)		14
14	Health care providers prescribing the mobile app in the clinic and asking about the compliance of the patients to use the app during the clinical visit	Advertisement or follow-up	4.00 (1.26)	5	8:3	6.91 (3.64)		3
15	Providing educational posters in the patient waiting area with a QR code to download the app	Advertisement	4.00 (1.18)	4	9:2	8.91 (4.87)		6
16	Providing services from trustworthy or well-known health care providers	Advertisement	3.82 (1.40)	4	9:2	9.18 (5.15)		4
17	Connecting the app with popular activity tracking devices (such as smartwatches, bands)	Features	3.82 (1.54)	4	9:2	12.73 (5.31)		11
18	Using colloquial terms in the push notification and information	Features	3.82 (0.87)	4	8:3	17.64 (2.20)		18
19	Providing inspirational and motivational quotes	Communication or support	3.64 (1.36)	4	8:3	13.09 (4.70)		10
20	Rewarding the users by offering nonfinancial incentives (such as gamification)	Cost	3.27 (1.74)	4	7:4	14.82 (5.09)		11
21	Rewarding the users by offering financial incentives	Cost	3.18 (1.78)	3	5:6	18.45 (3.50)		21

^a^The strategies are ordered in terms of the mean agreement score from round 3.

^b^Agreement rating, where 1=strongly disagree and 5=strongly agree.

^c^Ranking score, where 1=highest and 21=lowest.

^d^Agree: disagree is the ratio of “agree” and “strongly agree” to “neither,” “disagree,” and “strongly disagree,” which was used as an inclusion criterion for round 3.

## Discussion

### Features

In this study, participating diabetes experts expected many features to be included in a health app for people with diabetes. However, their level of agreement was slightly low, likely due to the variety of the experts’ backgrounds and the differing amounts of experience [29].

There is sufficient evidence from the literature supporting the effectiveness of the top features generated in this study, which are listed in descending order according to the level of rating agreement: (1) a reminder for the health care providers’ appointments, screening, and routine lab tests, which has been mentioned in several studies as a common use of health technology [30] that significantly affects outcomes [31]; and (2) carbohydrate counter and health diet planning**,** similar to tracking diary, carbohydrate, and meal intake [32,33] were recommended in several studies by diabetes educators who expressed their enthusiasm for viewing detailed dietary macronutrients [29]. The policy statement by the International Diabetes Federation (IDF) Europe uses some categories to differentiate between mobile health apps directed at people with diabetes. The first differentiating category was tracking, logging, and making dietary recommendations [34]. A qualitative study for weight and health management design indicates that the core components of the app should include tailored meal recommendations and assistance with meal planning [35]. For the nutrition education feature, a review found that the most useful mobile health app would help the patient with lifestyle education, self-management, and designing a suitable diet [36]. The IDF Europe categories also mention that such apps should help people with diabetes make food choices, undertake carbohydrate and calorie counting, and calculate medication dosages (similar to an insulin bolus calculator) [34]. A nutrition education app effectively raises awareness in people with diabetes [37].

For the contacts for guidance feature*,* the current National Health Service (NHS) services offer telephone contact points for youth. In addition, services with transition nurses, coordinators, or support workers offer contact via SMS text messaging [38]. The Saudi Arabian Ministry of Health (MOH) has a Service Center (that can be reached by dialing 937), which offers 24/7 medical doctor consultations [39] and can be added to mobile apps in Saudi Arabia. Several health apps suggested contacting HCP without providing specific contact details, access to experts, or just-in-time resources that could provide this type of guidance [40]. Other apps provided contact details for the diabetes health care team [41,42] or emergency contact lists [38].

For the “offering patient-specific education tailored to the user’s goals, their needs, and blood glucose readings” feature, end users appreciate that it saves time and provides instructions tailored to their specific condition [43]. Additionally, app designers should take into consideration context-sensitive details and condition information [33]. A study conducted to evaluate the effectiveness of diabetes apps discovered that 73% (11/15) of the apps on the market allowed the user to set goals and to visualize times they did not meet their goals, generally using a specific color to indicate hypoglycemia or hyperglycemia [32]. The app should facilitate goal setting (such as weight loss targets, fitness goals) [44] because diabetes is very individual; therefore, the app should be customizable [34]. Matching the participants' perspective, a study showed that participants wanted the medical app to provide information regarding health screening and functions that can assess their health; these must be personalized to them and trustworthy [45]. Targeted exploration of the literature found that action treatment plans and personalized health goals provided by HCP resulted in statistically significant outcomes in several studies [31].

Regarding the “finding the nearest urgent health care services/centers” feature, the app could detect the location of the user with the help of GPS and the global system for mobile communications (GSM) network, and thereby display information about the nearest medical centers. By clicking on a particular hospital, all information regarding that hospital could be provided; Furthermore, this is particularly advantageous for travelers as they could be connected with nearby health care centers if needed, which is a common concern for travelers with chronic diseases [46]. For the “database listing local restaurants and stores providing diabetes-friendly food” feature, a study modified a food database to provide symbolic food names and calorie information, including recipes, ingredients, and local food names [41], which can be added to the diabetes self-management health apps. Another study showed that 11 of 15 available diabetes management health apps on the market focused on tracking carbohydrate intake, while only 3 of 15 had a built-in food database [32]. One study showed that women with gestational diabetes liked that the illustrations of diet-related information could be customized to their culture [41].

For the “provision of Arabic and English versions” feature, a recent Chinese study showed that English- and Chinese-language diabetes self-management apps constitute more than 80% of the 2000 available diabetes apps. Furthermore, the Chinese and English apps have more downloads and are more comprehensive with regard to clinically relevant functions compared to diabetes apps in other languages [42]. A study showed that women with gestational diabetes had previously experienced challenges with care provision that involved the help of an interpreter; furthermore, translating medical terminology into other languages can be challenging [41]. To our knowledge, no study has evaluated the quantity and quality of Arabic diabetes self-management mobile apps.

For the “medication reminders by notifications” feature**,** this factor had a statistically significant relationship with the outcomes [31]. The IDF Europe has recommended that health apps should adopt SMS text messaging or push notifications for insulin injections. These reminders are essential as they could prevent hyperglycemia and long-term complications associated with prolonged uncontrolled diabetes [34].

For the “syncing with electronic medical records/personal health record*”* feature, a study showed that educators favored the integration of mobile phone–collected data into the health information system [29]. From the users’ perspective, a study showed that people with diabetes also wanted the app to be integrated into existing systems since it could help increase the dissemination of the app and improve app uptake [39]. For the “syncing with meters, continuous glucose monitors, or insulin pumps” feature, a study that surveyed youths with T1DM or their parents found that a glucometer-connected mobile app could increase an individual's engagement with other aspects of care (such as self-monitoring of blood glucose frequency) [47]. IDF Europe’s position on mobile apps for diabetes mentioned that interoperability is an essential feature of such apps [34].

### Engagement Strategies

Overall, there was a moderate degree of consensus among experts on their rankings of the engagement strategies that they believe are most likely to be effective in diabetes management when using a diabetic self-management health app. Strategies with the highest mean of agreement (ratings above 4.40) have already been addressed in the literature. Regarding the “app being free” feature, 36/48 (75%) of the included apps were free, and the average cost of the paid apps was Aus $4.37 (US $3.13) [48]. User ratings and prices are important factors determining app attractiveness, with variations across countries [49]. A study analyzing both free and paid apps found that more expensive and popular paid apps tended to have more drawbacks. This relationship between popularity and drawbacks emerges from the fact that more expensive and popular paid apps tend to have more functionality [50].

The importance of “allowing the user to communicate or send information/data to a health care provider” has been demonstrated at the patient and health care levels [34]. A thematic analysis

study focused on app-based interventions in managing chronic respiratory diseases, diabetes, and hypertension demonstrated the perceived ability of HCP-motivated patients and empowered them to properly self-manage their condition. The use of health technology in two-way communication between HCPs and patients proved to impact the patients’ health outcomes [31].

Studies found that user-friendly design*,* simplicity, and intuition were vital aspects for engaging younger adults to use health apps [34,51-53]. IDF Europe’s position on mobile apps for diabetes mentioned that determining the target audience is crucial for the uptake of an app [34].

Several studies have mentioned “flexibility and customization” as a requirement [29,54,55] and a key strategy to facilitate engagement with therapies. The ability to evaluate the app as a guest user, as well as the ability to modify the welcome message and color palate, are examples of customization and flexibility [56].

### Strengths and Limitations

One of the strengths of our study was having two people with diabetes on the study panel of experts. Therefore, besides their professional experience, they provided input into how the app could contribute to their life as patients with diabetes. The number of experts included was within the recommended range. Moreover, all the rounds were summarized and discussed by the steering committee, who are experts in the field.

There were several potential limitations identified in this study. First, we recruited experts with various backgrounds, which could have caused the significant levels of disagreement in the ranking. At the same time, if we had selected other experts, that could have led to a different result. Furthermore, there was considerable overlap among many of the information items volunteered by the Delphi participants. The use of a Delphi approach for selecting intervention components is not guaranteed to result in the best choices, which might affect the results’ reliability; this is a known limitation of the Delphi technique.

Another limitation is that our panel consisted only of experts. We did not include patients with diabetes, although two of the participants coincidentally had diabetes. To determine whether these same features and engagement strategies are desired by the end users, another validation study should be conducted among a population of people with diabetes to bridge the gap between the perspectives of experts and end users.

### Conclusions

To our knowledge, this is the first study in the Middle East and North Africa that gathered a local panel of experts from the diabetes field and used an iterative process to combine the experts’ opinions into a group consensus. Consensus agreement does not mean that all of the right answers have been found, but rather indicates that a level of participant agreement has been reached. The information items resulting from this modified Delphi survey represent the opinions of an expert panel.

This study allowed us to reach a consensus on several important questions related to diabetes management components that experts agreed were the most likely to be effective when delivered via a mobile app. Furthermore, it shed light on the engagement strategies that diabetes experts believed would be the most likely to be effective in diabetes management when using a mobile app. The results of this study could thus be useful for health authorities and HCPs for future decision making on this topic.

### Recommendations

The 36 features and 21 engagement strategies identified in this study should guide developers considering mobile app development targeting people with diabetes and other similar chronic diseases. For further research, we recommend that the results of this study should be verified and validated by building a prototype app that includes the features and strategies described in this study and recruiting people with diabetes to test it. Moreover, it would be more interesting to know if any of the top 10 app downloads globally or regionally contain these features and which combinations are most commonly found. How easily are these features implemented in an app? Are there cultural contexts that make certain feature more appropriate? Are any of the features not currently found in any app? These and other questions remain to be answered.

## Appendix

Multimedia Appendix 1 Intervention components generated by the experts in the first round.

Multimedia Appendix 2 Engagement strategies generated by the experts in the first round.

## Abbreviations

IDF: International Diabetes Federation
mHealth: mobile health
T2DM: type 2 diabetes mellitus
T1DM: type 1 diabetes mellitus
HCP: health care provider
