# Supplementary material for: Identification of Type 2 Diabetes Management Mobile App Features and Engagement Strategies: Modified Delphi Approach
Source: JMIR Mhealth Uhealth. 2020 Sep 11;8(9):e17083. doi: 10.2196/17083 (PMC7519429; doi:10.2196/17083)
Supplement: Multimedia Appendix 1 [file mhealth_v8i9e17083_app1.docx]

***Appendix 1: Intervention components generated by the experts in the first round***

***First question:***

Which features or components of management care delivered by mobile app would be best for helping people with diabetes improve their health?

***Medication:***

1. Medication reminders by notifications
2. Medication reminders by alerts
3. Medication reconciliation
4. Pill identifier
5. Description of diabetes medications (action and possible side effects and management)
6. Drug list name with detailed information
7. Assessing the medication adherence by validated Arabic scales
8. Adjusting insulin doses

***Physical activity:***

1. Individualized Medical plan for exercise management
2. Individualized exercise prescriptions
3. Give a medical advice based on the daily activity
4. Steps counter
5. Activity sensor tracker
6. Workouts and exercise demonstrations
7. Find the nearest health and fitness clubs

***Nutrition /Diet management:***

1. Carb counting
2. Nutrition Education
3. Food diary to track meal
4. Providing local low carb recipes.
5. Database for local restaurants and stores providing diabetes-friendly menus
6. Wight tracker
7. BMI tracker
8. BMI calculator
9. Health diet plans
10. Database for traditional low carb recipes
11. Database for food carbs and calories
12. Food barcode scanner
13. Meal picture detection to log food

***Home monitoring:***

1. Blood glucose monitoring diary
2. Reminder for frequent checking of Blood glucose
3. Reminders to check ketones level
4. Give a general advice in case of hyperglycemia and hypoglycemia.
5. Give customized advices based on the patient situation in case of hyperglycemia and hypoglycemia.
6. Saving the results (fasting, postprandial, random and HbA1c)
7. Give results for averages in graphs
8. Sharing blood glucose readings with healthcare professionals
9. Sharing blood glucose readings with family
10. Alert care giver (e.g. by text message) for abnormal/critical readings

***Follow-up care:***

1. Reminder for HbA1c testing
2. Reminder for the health care providers’ appointments
3. Easy downloading the results in the clinic.
4. Reminder for Eye screening
5. Reminder for Screening the issues that may impact DM: depression, mental status, hypogonadisms.
6. Medication and prescription refill reminders
7. Online consultation (communication and patient monitoring) by primary care providers
8. Reminder for routine lab tests, and other medical test.
9. Contacts for guidance if needed
10. Find the nearest urgent healthcare services/centers

***Psychosocial care:***

1. General advices regarding the psychological care
2. Customized advices regarding the psychological care
3. Using validated Arabic anxiety and depression scale
4. Tracking the patient symptoms and behavior and providing a positive message as notification
5. Brief education about common psychiatric symptoms associated with DM
6. Educate the family in case of hypoglycemia
7. Using validated Arabic anxiety and depression scales and giving customized advice to see a healthcare provider

***Education:***

1. information about diabetes in general
2. Information about diabetes and driving
3. Comprehensive teaching about insulin usage
4. Education about hypoglycemia and hyperglycemia management
5. Information about how to use the glucometer
6. Sick days management
7. Special occasion management during fasting, Hajj
8. Self management tips during travel
9. General advice about Foot care and Wound care
10. General advice about Dental care
11. Create personalized target goals
12. Offer patient-specific education tailored to the user’s goals, needs and blood glucose readings
13. The information should be recent evidence-based information
14. Alerts the user for personalized goals
15. General education about psychiatric symptoms associated with DM

***Mobile design/features:***

1. Sync with meters, CGMs, or insulin pumps.
2. Sync with electronic medical record/personal health record
3. Providing Arabic and English versions
4. Synchronization with personal health record (PHR)
5. Synchronization with Electronic health record (EHR)

***Social media and communication:***

1. Allowing for chat services for communication between users
2. Integrating the app with common social media channel platforms
