# Supplementary material for: Identification of Type 2 Diabetes Management Mobile App Features and Engagement Strategies: Modified Delphi Approach
Source: JMIR Mhealth Uhealth. 2020 Sep 11;8(9):e17083. doi: 10.2196/17083 (PMC7519429; doi:10.2196/17083)
Supplement: Multimedia Appendix 2 [file mhealth_v8i9e17083_app2.docx]

***Appendix 2: Engagement strategies generated by the experts in the first round.***

***Second question:***

What are the best strategies or techniques for engaging people with diabetes with a mobile app?

**Features:**

1. Efficient app
2. Using accessibility features (e.g. text-to-speech for people with limited vision)
3. Sync features
4. Recent Evidence based information
5. Using local term for the notification and information
6. Different teaching style (E.g. audiovisual, illustration, alarm and etc.)
7. Platform device syncing (cross-platform syncing (sync between mobile and other devices)
8. Connecting the app with popular activity tracking devices (e.g. smart watches, bands, etc.)
9. Connecting the app with the hospital information systems
10. Syncing with electronic medical records/personal health record

**Cost:**

1. Providing rewards to patients to use the app (if use it you will get free BS test device)
2. The app should be a free app
3. Rewarding the users by offering financial incentives
4. Rewarding the users by offering Non-financial incentives (e.g. Gamification)

**Easy to use:**

1. Ease of use.
2. Simple design
3. Friendly use.
4. Realistic alarming features
5. A user-friendly design
6. Educational and age-appropriate design
7. Flexibility of use

**Communication/ Support**

1. Allow the health care providers to communicate with their patients.
2. Direct contact with health care provider (Diabetes educator, Physician)
3. Capabilities to transfer data between the patient and health care providers
4. Provide Online counseling
5. Frequently asked questions section
6. Communication and patient monitoring by primary care providers
7. Providing a trustful health professional
8. Using colloquial terms in the push notification and information
9. Using different teaching methods (e.g. audiovisual, illustration, alarm etc.)
10. Allowing the user to communicate or send information/data to a healthcare provider
11. Allowing chat services for communication among users to support each other
12. Weekly supporting quotes about diabetes
13. Simple daily advices
14. Providing inspirational and motivational quotes
15. Allowing the users to share their progress with their family
16. Allowing the users to share their results with friends and family

**Personalized goals:**

1. Alert for personalized target for different variables an keeping notifying them.
2. Computable with PT age, level of education etc.
3. Personalized feedback

**Follow- UP:**

1. Ask about the apps in each clinic visit
2. Connect with the hospital appointment system
3. Diabetes calendar (patient can log their appointment, Fu examination, immunization status)
4. Provide the nearest health care facilities information in the living area
5. Taking feedback and adding new features

**Advertisement:**

1. Advertising and advocating the app by known health care providers
2. Providing services by trustful/known healthcare professionals
3. Prescribing the mobile app by healthcare providers in the clinic and asking about the compliance clinical visit
4. Advertising and advocating the apps by trustful healthcare associations
5. Providing educational posters in the patient waiting area with the app downloading QR codes
6. Explaining the usefulness of the app by health coaches on health campaigns
7. Recommending use of the app by official websites and social media accounts of scientific associations and organizations
